# *Anopheles* fauna of coastal Cayenne, French Guiana: modelling and mapping of species presence using remotely sensed land cover data

**DOI:** 10.1590/0074-02760160272

**Published:** 2016-11-10

**Authors:** Antoine Adde, Isabelle Dusfour, Emmanuel Roux, Romain Girod, Sébastien Briolant

**Affiliations:** 1 Institut Pasteur de la Guyane, Unité d’Entomologie Médicale, Cayenne, French Guiana; 2 Institut de Recherche pour le Développement, UMR ESPACE-DEV (University of French Guiana, University of French West Indies, University of la Réunion, University of Montpellier), Montpellier, France; 3 Direction Interarmées du Service de Santé en Guyane, Cayenne, French Guiana; 4 Institut de Recherche Biomédicale des Armées, Unité de Parasitologie et d’Entomologie Médicale, Hôpital d’Instruction des Armées Laveran, Marseille, France; 5 Faculté de Médecine La Timone, Unité de Recherche en Maladies Infectieuses Tropicales Emergentes, Marseille, France

**Keywords:** Anopheles, French Guiana, malaria, remote sensing technology

## Abstract

Little is known about the *Anopheles* species of the coastal areas of French Guiana, or their spatiotemporal distribution or environmental determinants. The present study aimed to (1) document the distribution of *Anopheles* fauna in the coastal area around Cayenne, and (2) investigate the use of remotely sensed land cover data as proxies of *Anopheles* presence. To characterise the *Anopheles* fauna, we combined the findings of two entomological surveys that were conducted during the period 2007-2009 and in 2014 at 37 sites. Satellite imagery data were processed to extract land cover variables potentially related to Anopheles ecology. Based on these data, a methodology was formed to estimate a statistical predictive model of the spatial-seasonal variations in the presence of *Anopheles* in the Cayenne region. Two *Anopheles* species, known as main malaria vectors in South America, were identified, including the more dominant *An. aquasalis* near town and rural sites, and *An. darlingi* only found in inland sites. Furthermore, a cross-validated model of *An. aquasalis* presence that integrated marsh and forest surface area was extrapolated to generate predictive maps. The present study supports the use of satellite imagery by health authorities for the surveillance of malaria vectors and planning of control strategies.

French Guiana is an overseas French territory of 250,000 inhabitants located in northern South America. Despite a continual decrease in the annual cases over the past decade (1.8 cases per 1,000 inhabitants in 2015), malaria remains a public health issue (Musset et al. 2014, Petit-Sinturel et al. 2016). *Anopheles darlingi* has historically been considered the main malaria vector in the territory, based on its natural infectivity, anthropophily, and broad distribution (Floch & Abonnenc 1943a, Pajot et al. 1977, Girod et al. 2008, 2011). However, among the other 23 *Anopheles* species present (Talaga et al. 2015), *An. nuneztovari s.l.*, *An. oswaldoi s.l.*, *An. intermedius*, *An. Marajoara*, and *An. ininii* have also been found naturally infected with *Plasmodium* sporozoites, but their exact implication in malaria transmission is yet to be clarified (Dusfour et al. 2012, Pommier de Santi et al. 2016a, b).

In the coastal area of Cayenne, the capital city of French Guiana, the antimalarial campaign of the early 1950s, which included widespread house-spraying and drug prophylaxis, resulted in the disappearance of *An. darlingi*, as well as a radical drop in malaria incidence (Floch 1954). Since then, the occasional cases that have been recorded in coastal areas have usually been imported from high prevalence locations (Carme et al. 2009). In particular, illegal gold miners are documented to travel regularly from endemic to malaria-free areas (Pommier de Santi et al. 2016c), and are highly suspected of contributing to the regular influx of exogenous parasites to coastal cities. If competent vectors are still present in these coastal areas, this influx might result in the local transmission and reintroduction of malaria to coastal areas (Cohen et al. 2012).

Owing to the threat of the reintroduction of malaria, public health authorities in coastal cities must be on constant alert, and effective oversight requires comprehensive knowledge of the parameters of malaria transmission, especially those involving vectors. However, little is known about *Anopheles* species in coastal areas, or their spatiotemporal distribution or environmental determinants. To the best of our knowledge, the last published sampling and mapping effort of *Anopheles* fauna in the Cayenne region (Fig. 1) dates to the late 1940s, before the antimalarial campaign. At that time, *An. aquasalis* was the most common species and was present at both near-ocean and inland sites. In decreasing order of frequency, *An. darlingi*, *An. braziliensis*, and *An. triannulatus* were also observed, although these three species were nearly absent at sites near the urban area of Cayenne.

**Fig. 1:**
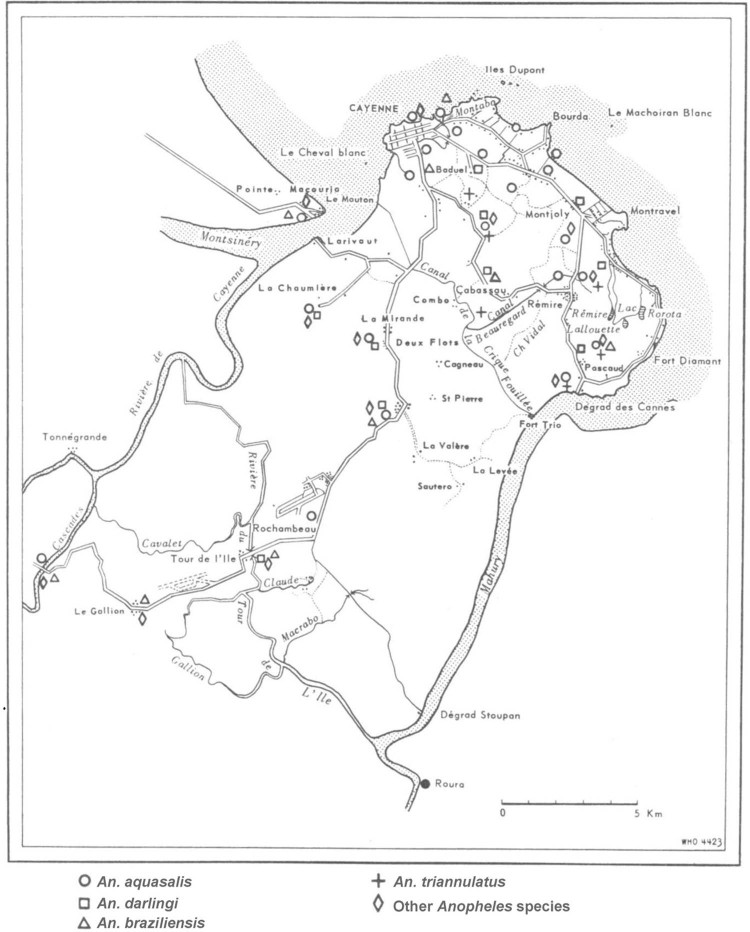
: historical distribution of *Anopheles* species in the coastal areas of Cayenne, French Guiana [adapted from Floch (1954)].

Accordingly, the aim of the present study was to furnish an updated map of the distribution of *Anopheles* species, using the findings of two entomological surveys, in order to assess the risk of reintroducing malaria to the coastal area of Cayenne. In addition, we also investigated the use of remotely sensed land cover data, in order to implement predictive models of *Anopheles* presence. The resulting maps and model of *Anopheles* occurrence may provide tools for future mosquito management and land-use planning.

## MATERIALS AND METHODS

*Geography of the study area* - The present study was conducted in the neighboring municipalities of Cayenne (4.94ºN, -52.33ºE), Rémire-Montjoly (4.90ºN, -52.28ºE), and Matoury (4.85ºN, -52.33ºE), which are the main industrial, political, and economic centers of French Guiana. The three cities encompass ~100,000 of the territory’s inhabitants, spread over a heterogeneous area of 200 km^2^ that includes a wide range of socioeconomic levels and natural environments. The climate of the study area is equatorial, hot, wet, and rainy. Daily mean temperatures range between 26.6ºC in January and 28.0ºC in October. Mean annual cumulative rainfall is 2,785 mm, with four alternating seasons: a long, wet season from April to mid-July; a long, dry season from mid-July to mid-November; a short, wet season from mid-November to mid-February; and a short, dry season from mid-February to the beginning of April.

**Fig. 2:**
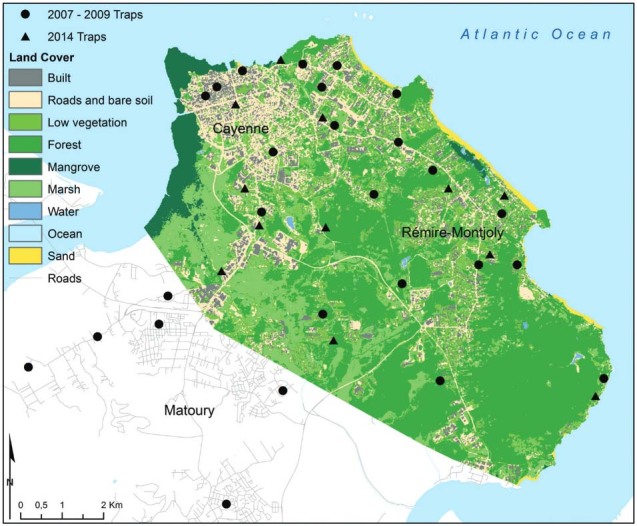
: land cover and *Anopheles* sampling sites, coastal areas of Cayenne, French Guiana.

*Mosquito collection* - Modelling and mapping of *Anopheles* species presence in the coastal area of Cayenne was achieved using the results of two entomological surveys that were conducted as part of this study over different sample periods, at different sample sites, and using different collection methods. The sampling sites were selected in order to maximise environmental heterogeneity (Fig. 2). For the first survey, adult *Anopheles* mosquitoes were collected by human landing catch at 25 different sites between November 2007 and October 2009. Each site was sampled on two consecutive evenings (6:00-8:00 pm) during one of the dry seasons, and two consecutive evenings during one of the wet seasons (Fig. 2). Mouth respirators were used to collect female mosquitoes that landed on the legs of consenting volunteer collectors, who had been informed of the associated risks. For the second survey, adult *Anopheles* mosquitoes were collected using Mosquito Magnet traps (Woodstream Co., Lititz, PA, USA) baited with octenol (MMoct) at 12 different sites. Each site was sampled on two consecutive evenings (6:00-8:00 pm) per week between April and June 2014 (wet season), and between September and November 2014 (dry season; Fig. 2). *Anopheles* mosquitoes were morphologically identified using taxonomic keys.

*Land cover characterisation* - A SPOT-5 image acquired on December 10, 2012 with four colour channels (red, green, near-infra-red, and middle infrared) at a 10-m spatial resolution was selected to characterise the land cover of the study area. However, because cloud cover prevented characterisation of the Matoury municipality, the mapping effort was restricted to Cayenne and Rémire-Montjoly; although a single image was sufficient to cover the two municipalities, the presence of residual clouds required the posterior use of a second SPOT-5 image (March 24, 2013) to fill in the missing data. Based on field observations, a supervised training approach with maximum likelihood classification was applied to produce a land cover map (Fig. 2) with nine land cover categories: built, roads and bare soil, low vegetation, forest, mangrove, marsh, beach, open water, and ocean. The quality of the final map was assessed by identifying the actual land cover of the training pixels, using photointerpretation, local field expertise, and cross-validation (Kappa coefficient = 0.88). Subsequently, surface areas for each land cover class were extracted from 100-, 200-, 300-, 400-, and 500-m radius buffers around each collection site. These radii were chosen to realise a compromise between a relevant landscape characterisation according to the satellite image spatial resolution, the overlap between neighbouring buffers (in order to avoid information redundancy and artificial spatial auto-correlation), and the MMoct constructor attraction radius (≥ 50 m). In total, 45 land cover variables (the surface areas of nine land cover classes from the five buffer sizes) were computed for each sampling site.

*Presence modelling and mapping* - The presence of *Anopheles* species in response to land cover was assessed using a generalised linear mixed model, lme4 R package version 1.1-12 (Bates et al. 2016) with a binomial distribution (presence or absence). As observations from the same collection point or season are likely to be more similar on average than observations from different collection points or seasons, these two components were considered explicitly through random effect terms in the model. Univariate models of *Anopheles* presence were fitted using each of the environmental features as explanatory variables, and environmental variables with p-values under 0.20 were retained for multivariate analyses. In the case of collinear explanatory variables, we selected the variable that maximised the log-likelihood and was the most ecologically relevant. Thereafter, we investigated all possible multivariate combinations of the remaining variables, and the selection of the final model was then based on statistical indicators, including the minimisation of Akaike information criterion (AIC) values (Akaike 1973), and maximisation of the area under the curve (AUC), which was computed using receiver operating characteristic (ROC) analysis. Model stability was assessed using a five-fold cross-validation, in which the cross-validated area under the curve (CVAUC) values were calculated as the average of the five AUC values of the refitted models. To map the results, the probability of *Anopheles* occurrence was calculated for each pixel of the study area, according to land cover composition, and then, based on the seasonal random intercepts of the model, both wet and dry season maps were produced. As land cover data were only available for the municipalities of Cayenne and Rémire-Montjoly, the Matoury municipality was excluded from the modelling and mapping procedures.

## RESULTS

**Fig. 3:**
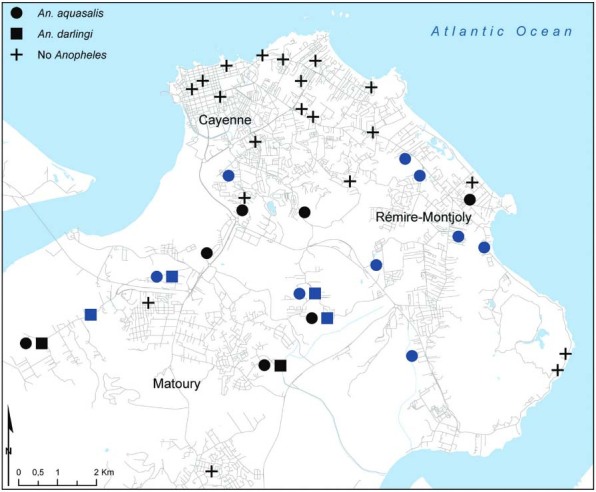
: distribution of *Anopheles* species in the coastal areas of Cayenne, French Guiana. Blue symbols indicate sampling sites at which the corresponding species were only found during the wet season.

*Distribution of Anopheles species* - Two *Anopheles* species were found: *An. aquasalis*, which was the most common and found at 16 of the 37 sites, and *An. darlingi*, which was only found at six of the sites (Fig. 3). No *Anopheles* species were collected at 20 of the sites and, for 10 of the sites, *Anopheles* species were only observed during the wet season. Spatially, *Anopheles* mosquitoes were mostly collected from the southern sampling points, which were outside the city centres. However, *An. aquasalis* was observed to occur over a broader geographic range than *An. darlingi*, being found at both near-town coastal sites and rural areas, whereas *An. darlingi* was only found at inland marsh and forest sites. In addition, at five of the six points where *An. darlingi* was found, *An. aquasalis* was also found. No *Anopheles* were collected in the northern sampling points in the urban area of Cayenne (Fig. 3).

*Predictive model of the An. aquasalis presence* - As *An. darlingi* was only found at two of the study sites where land cover data were available (i.e., in Cayenne and Rémire-Montjoly), the species was excluded from presence modelling. Thus, modelling and mapping efforts were focused on *An. aquasalis* alone. Univariate analysis of *An. aquasalis* presence and the 45 land cover variables yielded three noncollinear environmental variables with p-values lower than 0.20. These included (1) the surface area of marsh within a 400-m radius of the sampling points (Marsh400), (2) the surface area of forest within a 400-m radius around each of the sampling points (Forest400), and (3) the surface area of roads and bare soil within a 200-m radius of the sampling points (Bare200). The first two variables were positively associated and the third variable was negatively associated with *An. aquasalis* presence.

Following the AIC minimisation and AUC maximisation criterions, model 4 (Table I) was chosen as the final model. Indeed, this model, which was based on Marsh400 and Forest400, exhibited both the lowest and highest AIC (52.80) and AUC (0.93) values, respectively (Table II). The mean and standard deviation of the CVAUC values for the wet and dry season were 0.95 and < 0.02, and 0.89 and < 0.03, respectively, which were similar to those computed using the whole data set (0.96 and 0.89).

**TABLE I:** Statistical performance of land cover-based models of *Anopheles aquasalis* presence in the coastal areas of Cayenne, French Guiana

Model	Variables	AIC	AUC
1	Marsh400	59.70	0.87
2	Forest400	69.30	0.72
3	Bare200	71.10	0.70
4	Marsh400 + Forest400	52.80	0.93
5	Marsh400 + Bare200	56.60	0.90
6	Forest400 + Bare200	71.30	0.75
7	Marsh400 + Forest400 + Bare200	55.20	0.92

AIC: Akaike information criterion; AUC: area under the curve from the receiver operating characteristic (ROC) analysis.

**TABLE II:** Parameters of the final predictive land cover-based model of *Anopheles aquasalis* presence in the coastal areas of Cayenne, French Guiana

	Coefficient	Standard error	p-value
Intercept	-6.86	1.89	< 0.01
Slopes	-	-	-
Marsh400	-	-	-
Increase by one hectare	0.40	0.12	< 0.01
Forest400	-	-	-
Increase by one hectare	0.13	0.05	< 0.01
Random effects	-	-	-
Site	< 0.01	-	-
Season	1.04	-	-

**Fig. 4:**
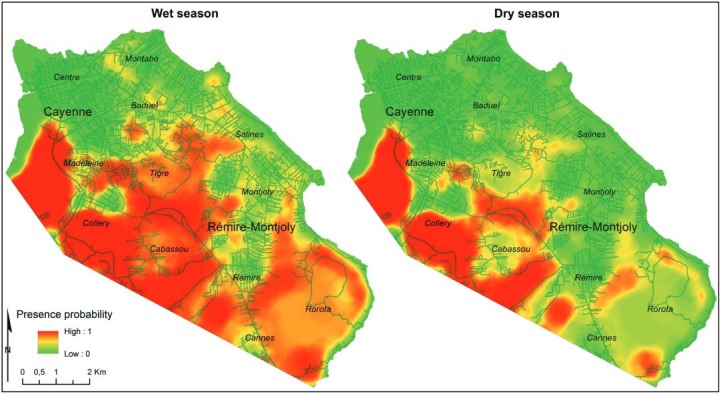
: probability of *Anopheles aquasalis* presence in the coastal areas of Cayenne, French Guiana.

*Predictive map of An. aquasalis presence* - Extrapolation of the model (Table II) allowed seasonal probability maps to be produced for *An. aquasalis* presence in the coastal area around Cayenne (Fig. 4). The spatial extent of the high probability area was largest during the wet season. In both dry and wet seasons, the high probability area encompassed the southern marshy and forested areas of the study area, including the industrial neighbourhood of Collery, as well as the residential areas of Madeleine, Tigre, and Cabassou. During the wet season, most of the coastal areas showed medium probabilities for *An. aquasalis* presence, but this was not the case during the dry season. The lowest probabilities were predicted at the city centres of Cayenne and Rémire-Montjoly, where forest and marsh patches were rare.

## DISCUSSION

To the best of our knowledge, the present study represents the first attempt to map the *Anopheles* fauna of the Cayenne region since the late 1940s (Fig. 1). We identified two *Anopheles* species that were reported in the last study (*An. aquasalis* and *An. darlingi*); however, *An. braziliensis* and *An. triannulatus* were no longer observed. The absence of these two species is probably attributable to the considerable environmental changes that have occurred over the area, namely urban sprawl, drainage of marshes, and deforestation (Gardel 2001).

In French Guiana, *An. darlingi* is strongly suspected to be responsible for the majority of malaria transmission (Floch & Abonnenc 1943a, Pajot et al. 1977, Girod et al. 2008, 2011). Although we failed to detect the species in urban neighbourhoods or near-coast sites, its presence was confirmed at the inland marsh and forest sites, as well as in the areas around Matoury, and in the neighbourhood of Cabassou. In these areas, which are documented as presumed sites of malaria contamination (Chocho et al. 2011), *An. darlingi* may ensure transmission. Interestingly, *An. darlingi* was previously incriminated in an epidemic in the early 1990s in Cabassou, following the settlement of immigrants in a formerly uninhabited area (Claustre et al. 2001). However, if *An. darlingi* can fuel transmission in localised foci, the scenario of malaria reintroduction to Cayenne is difficult to assess, owing to the species’ restricted spatial distribution.

As shown in historical entomological surveys (Floch & Abonnenc 1943b, Floch 1954, Floch & Fauran 1958, Pajot 1975, Claustre et al. 2001), the dominant *Anopheles* species in the littoral area of Cayenne was *An. aquasalis*. The species was found over a wider spatial extent than *An. Darlingi*, and only the sites located in the hyper-centre of Cayenne were free of *An. aquasalis*. *An. aquasalis* is known to be adapted to a broad range of ecological conditions (Grillet 2000), and is widely eclectic in its selection of breeding sites (Giglioli 1963). The species has been previously found in both brackish and fresh water, from coastal swamps to flooded forest, and most often in greater densities during the wet season (White 1951, Silvain & Pajot 1981, Berti et al. 1993a, Claustre et al. 2001).

In French Guiana, *An. aquasalis* has been historically depicted as having only a minor role in malaria transmission in the littoral area (Floch & Abonnenc 1943b, Pajot 1975, Silvain & Pajot 1981, Claustre et al. 2001). The involvement of *An. aquasalis* in malaria transmission in French Guiana has been disregarded, in part owing to the absence of individuals found naturally infected with *Plasmodium* sporozoites. Moreover, the weak vectorial capacity of the species, characterised by low longevity and zoophilic behaviour (White 1951, Giglioli 1963, Silvain & Pajot 1981, Berti et al. 1993b), may also contribute to the species’ inability to facilitate malaria transmission. However, specimens collected around Cayenne have shown successful infection experimentally (Floch & Abonnenc 1943b). In many other areas along the South American Atlantic coast, *An. aquasalis* has been known as the main vector of malaria (Giglioli 1963, Berti et al. 1993b, Grillet 2000), especially in Belém, Brazil, which is ~800 km south of Cayenne, where the species have been found to be naturally infected (Póvoa et al. 2003). Thus, it is possible that genetic differences in the *An. aquasalis* populations of French Guiana are responsible for the observed differences in vectorial capacity. However, in light of the species’ wide distribution around Cayenne and natural infectivity, *An. aquasalis* deserves regular monitoring.

In the present study, we also investigated the ecological preferences of *An. aquasalis* in the coastal area of Cayenne using a regression model and remotely sensed land cover data. As in the rest of the study, modelling efforts were focused on the presence rather than the density of species, in order to circumvent the biases associated with the different sampling methods used to build the final dataset. Nevertheless, it is clear that an unknown level of uncertainty remained, especially since human landing catches are more attractive to *Anopheles* mosquitoes than automatic traps (Vezenegho et al. 2014); however, the repetitive collections of the automatic traps (two consecutive nights per week for six months) suggest the method was as exhaustive as possible. In addition, species present in low densities or in unsampled areas may not have been identified.

The modelling results demonstrated that the presence of *An. aquasalis* in the coastal area of Cayenne is positively associated with the local (400-m radius) surface area of marsh and forest cover, and is reinforced during the wet season. The final model exhibited a high predictive value, with CVAUC values of 0.95 and 0.89 for the wet and dry seasons, respectively, and the resulting map provided valuable information regarding where and when vector control interventions should be focused. In addition, we also found that the environmental variables of the model could be used as proxies for predicting future *An. aquasalis* distributions in Cayenne under specific land use and land cover scenarios.

From a broader perspective, it would be pertinent to apply our method to other regions. Modelling and mapping approaches developed in the present study are not restricted to a specific context. Although new mosquito collections may be necessary to characterise local *Anopheles* fauna for undertaking such research, retrospective data from past studies can also be mobilised to extract new knowledge with less effort and cost.

Although the current rate of malaria transmission in coastal Cayenne is residual, regional health authorities must be constantly alert to the threat of reintroduction, owing to regular population influxes in endemic areas. Considering the observed and predicted distribution of *Anopheles* species, we conclude that the risk of reintroducing malaria to Cayenne and the surrounding areas is low, although it should still be monitored regularly. Thus, the present study demonstrates that geomatic tools, such as remote sensing, can be used to monitor the presence of malaria vectors *via* environmental proxies. In the future, public health authorities could use near real-time vector probability maps, produced using automated satellite data processing, as a valuable tool in planning more targeted and cost-effective mosquito control programs.
